# Dietary components and risk of total, cancer and cardiovascular disease mortality in the Linxian Nutrition Intervention Trials cohort in China

**DOI:** 10.1038/srep22619

**Published:** 2016-03-04

**Authors:** Jian-Bing Wang, Jin-Hu Fan, Sanford M. Dawsey, Rashmi Sinha, Neal D. Freedman, Philip R. Taylor, You-Lin Qiao, Christian C. Abnet

**Affiliations:** 1 Department of Cancer Epidemiology, Cancer Institute/Hospital, Chinese Academy of Medical Sciences & Peking Union Medical College, Beijing, 100021, China; 2 Nutritional Epidemiology Branch, Division of Cancer Epidemiology and Genetics, National Cancer Institute, Rockville, 20850, USA; 3 Department of Epidemiology and Health Statistics, School of Public Health, Zhejiang University, Hangzhou, 310058, China; 4 Genetic Epidemiology Branch, Division of Cancer Epidemiology and Genetics, National Cancer Institute, Rockville, 20850, USA

## Abstract

Although previous studies have shown that dietary consumption of certain food groups is associated with a lower risk of cancer, heart disease and stroke mortality in western populations, limited prospective data are available from China. We prospectively examined the association between dietary intake of different food groups at baseline and risk of total, cancer, heart disease and stroke mortality outcomes in the Linxian Nutrition Intervention Trials(NIT) cohort. In 1984–1991, 2445 subjects aged 40–69 years from the Linxian NIT cohort completed a food frequency questionnaire. Deaths from esophageal and gastric cancer, heart disease and stroke were identified through up to 26 years of follow-up. We used Cox proportional hazard models to calculate hazard ratios and 95% confidence intervals for associations between intake of groups of food items and these mortality endpoints. We concluded that higher intake of certain food groups was associated with lower risk of gastric cancer, heart disease and stroke mortality in a prospective cohort in rural China. Our findings provide additional evidence that increasing intake of grains, vegetables, beans, fruits and nuts may help reduce mortality from these diseases.

Diet is an important modifiable determinant of disease risk. Diet-related factors have been estimated to account for about 35% of cancers in developed countries^1^ and a lower proportion of cancers in developing countries, including an estimated 16% in China^2^. Previous studies have also shown that increased intake of some specific dietary food groups, such as grains, vegetables, and fruits, may protect against heart disease and stroke^3^,^4^. However, few epidemiological studies have examined the relationship between the intake of specific dietary components and risk of cancer, heart disease, or stroke in China^5^,^6^.

Linxian, a rural county in Henan Province, in north-central China, has some of the highest rates of esophageal and gastric cancer in the world^7^. The Linxian Nutrition Intervention Trials (NIT), including the Dysplasia Trial and the General Population Trial, were designed to test the efficacy of vitamin and mineral supplements in reducing the incidence and mortality of esophageal and gastric cancer. Recent data from the Linxian Dysplasia Trial suggested little benefit of multivitamin supplements on upper gastrointestinal cancers or total mortality^8^, but updated findings from the General Population Trial showed beneficial effect of factor D (selenium, vitamin E and beta-carotene) on gastric cancer and total mortality^9^. For this report, we analyzed prospective data from the NIT cohort to examine the association between diet components and risk of total, cancer, heart disease and stroke mortality in this rural population in China.

## Results

Table 1 summarizes the baseline demographic characteristics and frequency of consumption of the 16 food subgroup categories for the subset of the NIT cohort analyzed here. A total of 1104 men and 1341 women completed the detailed food frequency questionnaire. Consumption of tobacco and alcohol was very low among women in this cohort. Overall, diets were limited in scope and there was limited intake of fresh fruits and vegetables, meat/fish, and other sources of protein and relied heavily on non-whole grains. Women were less likely to have any intake of non-citrus fruits, starchy or liliaceae vegetables, red meat, nuts and eggs, but were more likely to consume whole grains than men.

During the 26-year follow-up period, 1501 deaths were identified, including 501 (33%) due to cancer, 355 (24%) due to heart disease, 452 (30%) due to stroke and 193 (13%) due to other causes. Of the 501 cancer deaths, there were 246 (49%) due to esophageal cancer and 175 (35%) due to gastric cancer, while the remainder occurred at variety of sites. In fully adjusted models, there were no significant associations between intake of any of the analyzed food groups and risk of total mortality or esophageal cancer mortality. However, a significant inverse association was observed for consumption of non-whole grains and gastric cancer mortality; increasing intake once/day decreased the risk by 14% (95% CI: 1%, 25%). A borderline inverse association was also seen for “other vegetables” and gastric cancer mortality; a once/day increase reduced the risk by 19% (95% CI: 0%, 34%; *P* = 0.054) (Table 2).

There was an overall decreased risk of heart disease mortality with higher baseline intake of vegetables or fruits. We found a HR of 0.89 (95% CI: 0.83, 0.96) for increasing total vegetable intake once/day, and significant associations also seen for the subgroups of yellow/orange vegetables and other vegetables, in which a once/day increase reduced the risk by 23% (95% CI: 3%, 40%) and 21% (95% CI: 7%, 32%), respectively. For fruit intake, we found a HR of 0.89 (95% CI: 0.82, 0.98) for increasing consumption of all fruits 3 times/month, and a HR of 0.93 (95% CI: 0.87, 0.99) for increasing consumption of non-citrus fruits by 2 times/month. We found significant inverse associations for higher intake of beans or nuts and risk of death from heart disease; increasing intake of beans 4 times/week and nuts 3 times/month reduced the risk by 37% (95 CI: 17%, 52%) and 11% (95 CI: 2%, 18%), respectively. In addition, we observed significant inverse associations between consumption of all grains, non-whole grains, and dark green vegetables and risk of stroke mortality (Table 2).

We also examined the associations between the food groups and our mortality outcomes by duration of follow-up, but these lag analysis did not alter any of our findings (data not shown). Finally, we performed analyses stratified by season of interview and trials (the Dysplasia Trial and the General Population Trial), but no differences across strata were found (data not shown).

## Discussion

This prospective study examined the associations between baseline intake of dietary food groups and subsequent risk of total, cancer, heart disease and stroke mortality. We found no significant associations between intake of any of the food groups and overall mortality or esophageal cancer mortality. Significant inverse associations were observed for mortality due to gastric cancer, heart disease, and stroke. We saw no evidence for significantly higher risk of mortality associated with greater intake of any of the food groups.

Total mortality has been examined in relation to diet in a number of observational studies. Studies of vegetarians and non-vegetarians indicate that vegetarians had lower total mortality than the general population^10^,^11^; meta- and combined analyses suggest similar findings^12^,^13^. A prospective cohort study of the European Prospective Investigation into Cancer and nutrition (EPIC) reported that the main components of the Mediterranean diet that predict lower total mortality are moderate intake of ethanol, low intake of meat, and high intake of vegetables, fruits, nuts, olive oil, and legumes^14^. A study of the Nordic diet found that fish, cabbage, rye bread, oatmeal, apples and pears, and root vegetables were associated with lower total mortality^15^. And a Chinese study reported lower mortality in persons with a fruit-rich diet that included apples, pears, peaches, oranges and other fruits^16^. Our study, however, did not identify significant associations between any of the food groups examined and total mortality.

Several previous studies have evaluated the association of dietary intake and cancer mortality^17^,^18^,^19^,^20^,^21^. A prospective study of over half a million people^17^ and a combined analysis of two cohorts^19^ indicated that red meat intake was associated with an elevated risk of cancer mortality. Two previous prospective studies also suggested that higher intake of fruits and vegetables were associated with lower risk of cancer death^20^,^21^. To our knowledge, few studies on diet and cancer mortality are available from Asian populations. In our study, we did not find significant associations between fruit and vegetable or meat intake and cancer mortality, except for a borderline inverse association between “other vegetables” and gastric cancer mortality. Possible explanations for these different findings include differences in study design, different populations, different food patterns and chance.

A previous study^22^ has evaluated the associations between a limited (11 item) list of dietary components hypothesized to be associated with health and risk of esophageal and gastric cancers in the Linxian General Population NIT cohort. The current study is a subset of that cohort and here we evaluated a more complete diet inventory of 64 food items. To assess the similarity of responses between the two questionnaires and to examine the representativeness of the subcohort, we used meat and egg consumption and compared the distribution of responses between the full NIT and the subcohort used in this analysis. We found the consumption of meat and eggs in this study was comparable with that in the entire General Population NIT cohort (data not shown).

Many studies have evaluated the relationship between dietary components and risk of cardiovascular disease-related mortality^23^,^24^,^25^,^26^,^27^,^28^,^29^,^30^,^31^,^32^. Most of these studies^23^,^24^,^25^,^26^,^27^,^28^ have focused on vegetables and fruits, and observed significant inverse associations between intake of these food groups and cardiovascular mortality. In our study, we found that higher consumption of fruits was associated with lower heart disease mortality, and higher consumption of dark green vegetables was associated with lower stroke mortality. Our results also suggest significant inverse associations for intake of nuts or beans and stroke mortality, and for intake of grains and heart disease death. Overall, our findings suggest that consuming more grains, fruit, vegetables, nuts and beans could reduce the risk of heart disease and stroke mortality in this rural Chinese population.

One potential concern in studies of diet and mortality is that dietary alterations may occur due to an illness before death. Hence, we performed lag analyses in which we excluded deaths that occurred during the first 13 years of follow-up. Our findings did not change, suggesting that baseline dietary patterns were not influenced by underlying preclinical disease.

The biological mechanisms underlying the associations between dietary components and risk of cancer, heart disease, and stroke remain unclear. Grains, vegetables and fruits are rich sources of many nutrients such as fiber, minerals (calcium, magnesium, selenium and zinc), vitamins (vitamins A, B, C and E), phenolic compounds, and antioxidants. These compounds have important functions and could have essential roles in decreasing the risk of cancer and cardiovascular disease. Several mechanisms have been proposed for the protective action of dietary fiber against cancer. Secondary bile acids promote cell proliferation, thus increasing replication of both normal and abnormal cells. But dietary fiber binds and dilutes primary bile acids, inhibiting the conversion of these primary bile acids to secondary bile acids. In addition, dietary fiber may protect against the risk of cardiovascular disease by affecting fibrinolysis and coagulation^33^.

Our study had several important strengths, including its prospective design, up to 26 years of follow-up, more than 1000 total deaths, and a broad range of food items evaluated. However it also had limitations. Exposure measurement error is always an issue in questionnaire-based dietary studies. In our study, individuals were asked how often they consumed foods during the past 3 months, but the portion size of each food was not collected. We were also unable to estimate or adjust for total energy intake. In addition, diet information was collected only at the baseline survey, and individuals may have changed their dietary practice during the 26-year follow-up. However, dietary patterns typically change gradually over time and most NIT subjects remain subsistence farmers. Although we had information on alcohol drinking, body mass index, and tobacco smoking, we lacked information on physical activity and some other lifestyle factors. As in other observational studies, our results could potentially reflect confounding by physical activity or uncontrolled lifestyle factors. However, all subjects in our study were enrolled from low socioeconomic level communities, and their lifestyles were very similar. Our findings could not be explained by the confounding effect of other lifestyle factors. Some of our significant results may have been due to the number of tests that we conducted that were not included in the pre-specified hypotheses. Furthermore, subjects in our study were selected from two nutritional intervention trials, which were conducted in parallel in Linxian, China. We were concerned that the source of subjects and the supplementation may have affected our results. However, we observed similar results after excluding participants from the dysplasia trial, and similar associations in these two trials among those who did and did not receive vitamins/minerals supplementation. Finally, our entire cohort was composed of rural Chinese adults, who consumed less meat, fish or eggs, included fewer overweight and obese individuals, and had fewer women smokers and drinkers than the general Chinese population. Therefore, there may be some limitations to the generalizability of our findings to other Chinese or non-Chinese populations.

In conclusion, in this prospective cohort study with 26 years of follow-up, beneficial effects were observed for mortality from gastric cancer, heart disease, and stroke from increased consumption of many different foods. Our findings suggest that increasing intake of grains, vegetables, beans, fruits and nuts may help reduce the risk of cancer and cardiovascular disease mortality.

## Methods

### Study population

Detailed information about the Linxian Nutrition Intervention Trials has been described previously^34^. Briefly, the Dysplasia Trial enrolled individuals with cytologically diagnosed esophageal dysplasia and was conducted in three communes in northern Linxian. Potential participants were eligible if they were between the ages of 40 and 69 years, lived in one of the three communes, provided written informed consent, and had a diagnosis of esophageal dysplasia based on a balloon cytology examination. A total of 3318 eligible residents were enrolled and were randomized to two groups receiving daily vitamin/mineral supplements or matching placebos for 6 years, beginning in May 1985. The vitamin/mineral supplements included 14 vitamins and 12 minerals in doses 2 to 3 times the US recommended daily allowances. The General Population Trial enrolled individuals from the general population of four communes in northern Linxian. Individuals were eligible if they were between 40 and 69 years old, lived in one of the four communes, and provided written informed consent. A total of 29,584 healthy adults were enrolled and randomized to eight intervention groups according to a one-half replicate of a 2^4^ factorial design, and they received daily vitamin/mineral supplementation for 5.25 years, beginning in March 1986.

In 1984–1991, 2445 NIT subjects from both trials (the Dysplasia Trial: 678 participants; the General Population Trial: 1767 participants) were randomly selected to complete an in-depth food frequency questionnaire during the supplementation period. We selected sufficient subjects to accurately characterize the local diet. Subsequently, this group was followed for 19–26 years, through the end of 2010, and total mortality and deaths from esophageal and gastric cancer, heart disease and stroke were identified. This study was approved by the Institutional Review Boards of US National Institutes of Health and the Chinese Academy of Medical Science, and all participants gave informed consent for the use of their blood samples and all data. All methods were carried out in accordance with the approved guidelines.

### Diet intake

The 64-item food frequency questionnaire (FFQ) contained items from 7 food categories, including 11 grain items, 32 vegetable items, 6 bean items, 16 fruit items, 3 nut items, 5 meat and fish items and 1 egg item (Table 3). For each item, participants were asked how often, on average, they consumed these foods during the 3 months before the interview date. The possible responses ranged from “never” to “several times per day”. The responses were then converted to different units according to the distribution of the data, e.g. times per day for grains, times per week for vegetables and beans, and times per month for other food items. We also combined specific food items into 16 subgroups similar to those used in other diet studies but adapted to Chinese food patterns^35^, including non-whole grains, whole grains, dark-green vegetables, yellow/orange vegetables, starchy vegetables, cruciferous vegetables, liliaceae vegetables (chives and scallions), other vegetables, beans, citrus/melon fruits, non-citrus fruits, nuts, red meat, white meat, fish and eggs (Table 3).

### Outcomes

The main endpoints for the study were total deaths, esophageal cancer deaths, gastric cancer deaths, heart disease deaths, and stroke deaths. During the trial period (1985–1991), village doctors visited participants monthly, trial staff periodically reviewed local and regional hospital records and the local cancer registry for records of deaths and cause of death among the participants, and a panel of American and Chinese experts reviewed and verified 85% of the cancer cases. During the subsequent post-trial period, all living participants were contacted monthly, and a panel of Chinese experts verified new cancer cases and all causes of deaths. Diagnostic materials used in these expert reviews included case records, pathology and cytology slides, X rays, biochemical results, and ultrasound, endoscopy and surgery reports.

### Statistical analysis

Our analyses focused on the associations between 14 food subgroups (red meat, white meat and fish were combined as one subgroup) and total, cancer, heart disease, and stroke mortality. Follow-up time started after the completion of the food questionnaire between 1984 and 1991. Participants were censored at their last known follow-up date, death, or December 31, 2010, whichever came first. All diet items were analyzed as continuous variables (times/day, times/week, or times/month). For our primary analysis, we estimated hazard ratios (HR) and 95% confidence intervals (CI) using Cox proportional hazard models adjusted for age (continuous variable), sex (male or female), commune (Yaocun, Rencun, Donggang, or Hengshui), smoking [yes (regular cigarette or pipe use for at least six months) or no], drinking [yes (any consumption of alcohol in the previous 12 months) or no], season of interview [spring (March–May), summer(June–August), autumn (September–November) or winter (December–February)] and BMI (continuous variable). All HRs and 95% CI were scaled to the interquartile range of food subgroups and average food consumption frequencies in local areas. We tested the assumption of proportional risk for the Cox models by using cross-product terms (time × food item). If a cross-product term was statistically significant, we examined Kaplan-Meier curves to assess the proportional hazard assumption, and if there appeared to be a difference in association by follow-up duration, we performed other analyses stratified by follow-up time. We also performed analyses stratified by season of interview. All p-values were two-sided, and p-values <0.05 were considered statistically significant. All statistical analyses were performed using SAS 9.2 (SAS Institute, Inc, Cary, NC).

## Additional Information

**How to cite this article**: Wang, J.-B. *et al.* Dietary components and risk of total, cancer and cardiovascular disease mortality in the Linxian Nutrition Intervention Trials cohort in China. *Sci. Rep.* **6**, 22619; doi: 10.1038/srep22619 (2016).

## Figures and Tables

**Table 1:** Baseline demographic characteristics and frequency of consumption of 16 food categories by sex in the analyzed subset of the Linxian Nutrition Intervention Trials, Linxian, China.

	All participants	Men	Women
**No. of participants (n, %)**	2445 (100.0)	1104 (45.2)	1341 (54.8)
**Age groups (n, %)**			
<50	664 (27.2)	239 (21.6)	425 (31.7)
50–59	909 (37.2)	398 (36.1)	511 (38.1)
≥60	872 (35.6)	467 (42.3)	405 (30.2)
**Smoking (n, %)**			
Non-smoker	1688 (69.1)	352 (31.9)	1336 (99.8)
Smoker	754 (30.9)	752 (68.1)	2 (0.2)
**Alcohol drinking (n, %)**			
Non-drinker	1888 (77.3)	682 (61.8)	1206 (90.1)
Drinker	554 (22.7)	422 (38.2)	132 (9.9)
**BMI (Median, IQR)**	21.3 (19.8, 23.0)	21.2 (19.9, 22.6)	21.5 (19.8, 23.4)
**Grains, times/day (Median, IQR)**			
Non-whole	3.4 (2.8, 4.5)	3.4 (3.0, 4.6)	3.4 (2.7, 4.5)
Whole	2.0 (0.2, 2.0)	1.2 (0.1, 2.0)	2.0 (0.3, 2.0)
**Vegetables, times/week (Median, IQR)**			
Dark green	0.0 (0.0, 2.0)	0.0 (0.0, 2.0)	0.0 (0.0, 2.0)
Orange	2.0 (0.0, 7.0)	2.0 (0.0, 7.0)	2.0 (0.0, 7.0)
Starchy	2.2 (0.5, 7.0)	2.7 (0.5, 7.0)	2.2 (0.5, 7.0)
Cruciferous	0.0 (0.0, 7.0)	0.0 (0.0, 7.0)	0.2 (0.0, 7.0)
Liliacae	2.0 (0.0, 7.0)	2.0 (0.0, 7.0)	1.0 (0.0, 7.0)
Others	5.2 (2.5, 9.2)	5.2 (2.5, 9.1)	5.4 (2.5, 9.5)
**Beans, times/week (Median, IQR)**	2.0 (0.7, 5.0)	2.0 (0.7, 5.0)	2.0 (0.5, 5.0)
**Fruits, times/month (Median, IQR)**			
Citrus/melon	0.0 (0.0, 0.7)	0.0 (0.0, 1.0)	0.0 (0.0, 0.7)
Non-citrus	0.3 (0.0, 2.0)	0.7 (0.0, 2.3)	0.0 (0.0, 2.0)
**Nuts, times/month (Median, IQR)**	0.3 (0.0, 3.0)	0.5 (0.0, 4.3)	0.0 (0.0, 2.0)
**Meat and fish, times/month (Median, IQR)**			
Red meat	0.7 (0.0, 2.0)	1.0 (0.0, 2.0)	0.3 (0.0, 2.0)
White meat	0.0 (0.0, 0.0)	0.0 (0.0, 0.0)	0.0 (0.0, 0.0)
Fish	0.0 (0.0, 0.0)	0.0 (0.0, 0.0)	0.0 (0.0, 0.0)
**Eggs, times/month (Median, IQR)**	1.0 (0.3, 4.3)	2.0 (0.7, 4.3)	1.0 (0.0, 4.3)

IQR, Interquartile Range.

**Table 2:** Hazard ratios (HR) and 95% confidence intervals (CI) for the associations between baseline consumption of different food groups and specific causes of death in the Linxian Nutrition Intervention Trials, Linxian, China (1984–2010).

Food groups	Scale#	HRs (95 % CI)*				
		Total death (N = 1501)	Esophageal cancer (N = 246)	Gastric cancer (N = 175)	Heart disease (N = 355)	Stroke (N = 452)
Grains						
All	Once/day	0.96 (0.93, 1.00)	0.93 (0.84, 1.03)	0.93 (0.82, 1.05)	1.00 (0.92, 1.09)	**0.90 (0.83, 0.97)**
Non-whole	Once/day	0.96 (0.92, 1.01)	0.94 (0.83, 1.06)	**0.86 (0.75, 0.99)**	1.05 (0.94, 1.16)	**0.90 (0.82, 0.98)**
Whole	Once/day	0.98 (0.93, 1.05)	0.94 (0.81, 1.09)	1.08 (0.91, 1.29)	0.94 (0.83, 1.07)	0.93 (0.83, 1.04)
Vegetables						
All	Once/day	0.98 (0.95, 1.01)	0.99 (0.91, 1.08)	0.95 (0.85, 1.05)	**0.89 (0.83, 0.96)**	1.01 (0.95, 1.07)
Dark green	twice/week	0.88 (0.73, 1.07)	1.07 (0.68, 1.69)	1.08 (0.64, 1.80)	0.72 (0.48, 1.06)	**0.62 (0.43, 0.91)**
Yellow/orange	Once/day	0.95 (0.86, 1.06)	0.97 (0.74, 1.27)	0.96 (0.70, 1.33)	**0.77 (0.60, 0.97)**	0.92 (0.76, 1.12)
Starchy	Once/day	1.01 (0.90, 1.18)	0.95 (0.74, 1.24)	1.01 (0.74, 1.38)	0.95 (0.76, 1.20)	1.16 (0.96, 1.41)
Cruciferous	Once/day	1.03 (0.95, 1.17)	1.09 (0.79, 1.51)	1.05 (0.70, 1.58)	0.81 (0.60, 1.11)	1.06 (0.83, 1.34)
Liliacae	Once/day	1.05 (0.93, 1.19)	0.93 (0.67, 1.27)	0.93 (0.63, 1.35)	0.98 (0.75, 1.28)	1.17 (0.93, 1.47)
Other†	Once/day	0.93 (0.87, 1.00)	0.99 (0.85, 1.16)	**0.81 (0.66, 1.00)**	**0.79 (0.68, 0.93)**	1.01 (0.89, 1.14)
Beans	4 times/week	0.90 (0.81, 1.01)	1.11 (0.87, 1.42)	1.05 (0.77, 1.42)	**0.63 (0.48, 0.83)**	0.84 (0.68, 1.04)
Fruits						
** **All	3 times/month	0.99 (0.96, 1.02)	1.01 (0.96, 1.06)	1.04 (0.99, 1.10)	**0.89 (0.82, 0.98)**	0.98 (0.93, 1.04)
** C**itrus/melon	Once/month	0.98 (0.95, 1.00)	0.99 (0.94, 1.05)	1.00 (0.94, 1.06)	0.92 (0.84, 1.01)	0.96 (0.90, 1.03)
** **Non-citrus	2 times/month	1.00 (0.98, 1.02)	1.01 (0.97, 1.06)	1.05 (1.00, 1.10)	**0.93 (0.87, 0.99)**	0.99 (0.95, 1.04)
Nuts	3 times/month	0.99 (0.96, 1.02)	1.00 (0.93, 1.08)	0.98 (0.89, 1.07)	**0.89 (0.82, 0.98)**	0.99 (0.93, 1.05)
Meat/fish^**&**^	2 times/month	1.00 (0.97, 1.02)	0.98 (0.92, 1.05)	1.03 (0.97, 1.09)	0.94 (0.88, 1.02)	1.02 (0.97, 1.07)
Eggs	4 times/month	1.01 (0.98, 1.03)	0.99 (0.92, 1.06)	1.04 (0.97, 1.10)	1.00 (0.95, 1.06)	1.00 (0.96, 1.06)

^#^HRs scaled to Once/day for grains and vegetables (except for dark green vegetables, twice/week), 4 times / week for beans, several times/month for fruit, nuts, meat/fish and eggs.

^*^Adjusted for age, sex, commune, smoking, drinking, season and body mass index.

^†^†Other vegetables included fresh radish roots, fresh turnip root or mustard roots, garlic bulbs, celery, tomatoes, fresh eggplants, sweet green peppers, cucumbers, winter melons, dried eggplants, dried radish roots, dried turnip roots and mustard roots.

^&^Combined red meat, white meat and fish as one subgroup.

**Table 3:** Food groups in the food frequency questionnaire of the Linxian Nutrition Intervention Trials, Linxian, China.

Groups	Subgroups	Description
**Grains**		
	Non-whole grains	Wheat flour steamed bread
		Wheat flour noodles
		Wheat flour pancakes
		Rice
		Millet grain
		Millet chaff & persimmon bread
	Whole grains	Corn meal porridge
		Corn meal steamed bread
		Corn meal flat cakes
		Corn meal dumplings
		Sorghum
**Vegetables**		
	Dark-Green	Spinach
		Fresh sweet potato leaves
		Fresh radish or mustard greens
		Chinese parsley (coriander)
		Dried sweet potato leaves
		Dried greens
	Yellow/orange	Fresh sweet potatoes
		Carrots
		Orange winter squash or pumpkins
		Pale green squashes or gourds
		Dried sweet potatoes
	Starchy	White potatoes
		Fresh green peas or beans
		Fresh corn on the cob
		Dried white potatoes
	Cruciferous	Chinese cabbage, or other cabbages
		Dried Chinese cabbage, or other cabbages
		Cauliflower
	Liliaceae	Chives or scallions
	Others	Fresh radish roots
		Fresh turnip root or mustard roots
		Garlic bulbs
		Celery
		Tomatoes
		Fresh eggplants
		Sweet green peppers
		Cucumbers
		Winter melons
		Dried eggplants
		Dried radish roots
		Dried turnip roots, mustard roots
**Beans**	Beans	Cooked dried soy bean
		Any other kind of cooked dried beans or peas
		Fresh bean curd
		Dried bean curd
		Fermented. salted pickled bean curd
		Bean sprouts
**Fruits**		
	Citrus/melons	Any kind of sweet melons
		Chinese hawthorn
		Oranges
	Non- Citrus	Apples or pears
		Peaches
		Fresh grapes
		Dried grapes
		Fresh apricots
		Dried apricots
		Plums
		Fresh persimmons
		Dried persimmons
		Fresh dates
		Dried dates
		Fresh litchis
		Dried litchis
**Nuts**	Nuts	Peanuts
		Chestnuts
		Walnuts
**Meat and fish**		
	Red meat	Pork in any form
		Beef in any form
		Liver of any kind
	White meat	Chicken or duck
	Fish	Fish or shellfish in any form
**Eggs**	Eggs	Eggs, either fresh or preserved
